# Exploring Emotional Self-Efficacy as a Mediator of Positive Leisure Experience and Subjective Well-Being Among Elementary School-Age Children in a Marginalized Community

**DOI:** 10.3390/healthcare13222982

**Published:** 2025-11-20

**Authors:** Mei-Ling Lin

**Affiliations:** Department of Occupational Therapy, School of Health Professions, The University of Texas at San Antonio, San Antonio, TX 78229, USA; linm2@uthscsa.edu; Tel.: +1-210-450-8378

**Keywords:** emotional self-efficacy, leisure enjoyment, life satisfaction

## Abstract

**Background**: Prior research has established a positive relationship between emotional self-efficacy and life satisfaction in elementary school-age children. However, less is known about the direct impact of positive leisure experience on subjective well-being and the potential mediating role of emotional self-efficacy. **Objectives**: This study examined whether emotional self-efficacy mediates the association between overall leisure enjoyment and life satisfaction among elementary schoolchildren. It was hypothesized that both direct and indirect effects are statistically significant. **Methods**: A quantitative, cross-sectional design was used with 100 fifth- and sixth-grade students from a U.S.–Mexico border community. Participants completed the Children’s Assessment of Participation and Enjoyment (CAPE), the emotional subscale of the Self-Efficacy Questionnaire for Children (SEQ-C), and the Student Life Satisfaction Scale (SLSS). Mediation analysis was conducted in R with bootstrapping (500 simulations). **Results**: Overall leisure enjoyment was positively associated with life satisfaction (β = 0.54, 95% CI [0.23, 0.90], *p* = 0.004). The direct effect remained significant after accounting for emotional self-efficacy (β = 0.41, 95% CI [0.15, 0.73], *p* = 0.004). The indirect effect through emotional self-efficacy was also significant (β = 0.13, 95% CI [0.03, 0.29], *p* = 0.016), accounting for approximately 25% of the total effect. **Conclusions**: Emotional self-efficacy partially mediated the relationship between overall leisure enjoyment and life satisfaction, suggesting that positive leisure experience enhances children’s emotional coping confidence and subjective well-being. These findings underscore the importance of promoting accessible and enjoyable leisure opportunities within marginalized communities that simultaneously foster children’s emotional self-efficacy and well-being.

## 1. Introduction

A typical day for an elementary school-age child encompasses a range of activities, including self-care (e.g., dressing, grooming, feeding and eating, functional mobility), education, social participation, play, and leisure exploration [1]. During the weekdays, elementary schoolchildren typically spend approximately six hours per day in formal educational settings, where they acquire academic knowledge, develop social–emotional competencies, and utilize sensorimotor skills to address basic needs, play, and navigate the school environment safely. Following the school day, many children participate in extracurricular or community-based programs that facilitate the exploration of personal interests and support continued social engagement. Others return home, where daily routines may involve unstructured play or leisure activities under the supervision of caregivers. Evening routines generally include mealtimes, bathing or showering, grooming, and preparation for rest and sleep. The composition of a child’s daily activities is shaped by multiple contextual factors, including age and developmental level, family values and educational preferences (e.g., homeschooling vs. traditional schooling), household socioeconomic status (e.g., affordability and accessibility of extracurricular opportunities), neighborhood and community resources ([2]; e.g., availability of safe and supportive play environments), and cultural practices ([3,4]; e.g., emphasis on family and relative caregiving in Hispanic communities).

When children are able to fully participate in a balanced range of activities, they are more likely to experience positive physical and mental health outcomes, while also developing increasingly sophisticated physical, social–emotional, and cognitive abilities. For children growing up in marginalized communities, opportunities for safe, engaging, and enjoyable leisure may be limited by socioeconomic barriers and environmental constraints [5,6]. Such disparities not only restrict access to enriching experiences but also increase vulnerability to poorer physical and mental health outcomes.

The present study uniquely recruited children residing in a US–Mexico border region, a marginalized community, and specifically investigated the direct association between children’s perceived enjoyment of leisure activities and life satisfaction and evaluated the mediating role of emotional self-efficacy in this relationship. The findings are expected to inform practices that advance equity in child health and development by identifying the role of enjoyable leisure experiences (the leisure pursuits that children perceive as pleasurable, satisfying, or emotionally rewarding) in supporting mental well-being.

### 1.1. Multidimensional Benefits of Enjoyment in Leisure Activity

Leisure activities refer to non-obligatory, intrinsically motivated activities that occur outside school and home responsibilities and are freely chosen by the child [1]. The majority of scholarly inquiries into children’s leisure activities have centered on physical activity. Enjoyment is a critical factor in sustaining regular participation in physical activity, which is essential for promoting an active lifestyle and achieving long-term health benefits [7]. From a physical health standpoint, engaging in physical activity supports the development of strong bones, muscles, and joints, while also reducing the risk of chronic conditions such as obesity and Type 2 diabetes [8,9]. Consistent participation enhances muscular strength, cardiovascular endurance, and motor coordination, thereby improving overall physical fitness [10].

In addition to physical advantages, regular participation in physical activities provides significant mental and emotional benefits. These include increased self-esteem, improved attention and memory, better academic performance, and enhanced overall psychological well-being [11]. Socially, physical activities, especially those conducted in group settings, offer valuable opportunities for social interaction, cooperation, and the development of communication and problem-solving skills [10,12]. Thus, enjoyment not only encourages ongoing participation but also underpins the physical, mental, and social benefits associated with an active lifestyle [13].

Although enjoyment has been recognized as a key motivator for sustaining physical activity among Hispanic children [14], research exploring their subjective experiences of enjoyment during physical activity remains limited. Furthermore, participation in and enjoyment of other forms of leisure activities—such as fine arts, cognitive games like chess, and other non-physical pursuits—have been significantly understudied within this population. Lastly, the potential relationship between perceived level of enjoyment in all leisure activities and broader indicators of well-being, has yet to be empirically examined.

### 1.2. Children’s Activity Participation and Enjoyment (CAPE)

The Children’s Assessment of Participation and Enjoyment (CAPE) is a widely used tool in empirical research to evaluate children’s participation in leisure activities. It measures both the diversity and intensity of activity participation, as well as the enjoyment associated with these activities. Specifically, CAPE categorizes leisure activities into five domains [15]:

Recreational activities refer to informal, self-directed pursuits primarily undertaken for relaxation or amusement, such as watching television, listening to music, or playing with toys.

Physical activities include those requiring bodily movement or exertion, such as running, swimming, cycling, or playing sports.

Social activities encompass interactions with peers, family, or community members, including talking on the phone, visiting friends, or participating in group games.

Skill-based activities involve structured opportunities to acquire or refine specific abilities through practice or instruction—examples include art lessons, dance classes, musical instrument practice, or organized club participation.

Self-improvement activities are aimed at personal growth or cognitive engagement, such as reading for pleasure, doing homework, computer use for learning, or participating in community or religious events.

Together, these domains capture the multifaceted nature of children’s leisure experiences, spanning passive and active, individual and social, and physical and cognitive forms of engagement. The inclusion of all five domains allows for a comprehensive understanding of how different types of leisure contribute to children’s overall enjoyment and well-being.

CAPE has been adapted and validated in multiple languages and cultural contexts, demonstrating its reliability and applicability across diverse populations, such as Portuguese children [16,17]. Cross-cultural validation studies have further supported its construct validity, showing positive relationships between physical well-being and overall frequency of leisure participation [17].

CAPE has also been applied to examine how leisure activity participation relates to children’s developmental outcomes, such as motor performance and psychological well-being. For example, Esposito et al. [18] investigated participation patterns among typically developing Dutch children aged 6 to 8 years and found that higher participation diversity and intensity were associated with stronger motor performance, including manual dexterity and balance. Similarly, Cho [19] reported that enjoyment in physical activity positively influences Korean children’s self-efficacy and intention to engage in physical activities. Notably, self-efficacy partially mediated the effect of enjoyment, suggesting that children who find physical activities enjoyable not only develop greater confidence in their abilities but are also more motivated to continue participation.

### 1.3. Patterns of Leisure Participation in Hispanic Children

Participation in physical activities has received the most scholarly attention among various types of leisure engagement in children and youth. For Hispanic school-age children, patterns of leisure participation reflect a complex interplay of demographic, cultural, and environmental factors that shape their physical activity levels. Research has documented significant disparities in physical activity engagement within this population, with important implications for addressing obesity and promoting healthier lifestyles.

Participation in physical education (PE) and extracurricular sports is a critical determinant of children’s physical activity levels. However, many Hispanic children—particularly girls—demonstrate limited engagement in these opportunities, which correlates with lower physical activity levels [20]. Compared to their non-Hispanic peers, Hispanic children are less likely to participate in organized sports. This disparity is especially pronounced in rural communities, where structural and socioeconomic barriers limit access to sports programs [21,22]. Additionally, physical activity levels tend to decline with age among Hispanic youth, with boys generally being more active than girls [23].

Collectively, these findings highlight the dual role of enjoyment in children’s activity participation. Enjoyment directly strengthens children’s intention to participate in physical activities, while simultaneously enhancing self-efficacy, which in turn reinforces ongoing participation. This interplay underscores the importance of fostering positive and enjoyable activity experiences to support children’s health and well-being.

### 1.4. Theoretical Framework: The Broaden-and-Build Theory of Positive Emotions

Fredrickson’s broaden-and-build theory of positive emotions [24] provides a useful framework for understanding how leisure enjoyment may enhance children’s emotional self-efficacy and subjective well-bring. According to this theory, positive emotions such as joy, interest, and contentment temporarily broaden individuals’ thought–action repertoires, encouraging exploration, creativity, and social connection. Over time, these repeated moments of broadened cognition and behavior build enduring psychological resources, including emotional resilience and coping capacities. When children engage in enjoyable leisure activities, the positive emotions elicited during participation can therefore expand their awareness, foster adaptive coping strategies, and promote a sense of mastery in regulating emotions. Through these processes, leisure enjoyment may contribute to the gradual development of emotional self-efficacy, which in turn supports greater subjective well-being. This theoretical perspective offers a coherent explanation for the hypothesized mediational pathway linking leisure enjoyment, emotional self-efficacy, and life satisfaction.

### 1.5. Study Objectives and Hypothesis

This study is part of a broader cross-sectional research project focused on understanding mental health and subjective well-being among children from ethnic minority backgrounds. Prior findings from the project indicated a positive association between emotional self-efficacy and life satisfaction [25]. Building on this, the current study aimed to investigate the direct effect of overall leisure enjoyment on perceived life satisfaction, as well as the potential mediating role of emotional self-efficacy in this relationship.

Drawing on the broaden-and-build theory of positive emotions [24] and Cho’s findings [19] that enjoyment in physical activity can positively influence self-efficacy, it was hypothesized that overall leisure enjoyment would have both a direct effect on life satisfaction and an indirect effect mediated through emotional self-efficacy. Both effects were expected to be statistically significant.

## 2. Materials and Methods

### 2.1. Research Design

This study employed a quantitative, cross-sectional design to examine the direct and indirect effects of overall leisure enjoyment on life satisfaction using data collected from one elementary school located in one US–Mexico border community in Texas. The current study was approved by the Institutional Review Boards of the investigator’s affiliated university at the time of study period and the partnering school district. Both parent permission and child assent forms were obtained prior to any research activity.

### 2.2. Study Setting and Participant Recruitment

The study was conducted between September to December 2019. According to the school district’s report on the district students’ academic performance during the 2018–2019 school year, 87% of fifth graders and 69% of sixth graders’ reading performance approaches the grade level or above. According to the school website, 71% were from households that have been economically/socially marginalized, 88% were from Hispanic origins, and 26% were English language learners.

Using convenience sampling, all parents or guardians of fifth and sixth graders (*n* = 249) were informed about the research opportunity for their child to participate. After excluding students who did not obtain parental consent or were unable to comprehend and respond to the survey questions in either English or Spanish, a total of 100 children voluntarily participated. The final sample included 39 boys and 60 girls (with one participant missing gender data), comprising 43 sixth graders and 57 fifth graders.

After signing the assent forms, participants were asked to complete three questionnaires in their designated space in the school gymnasium. The school counselor, nurse, teachers, and researchers were actively providing language support (e.g., explaining terms and reading questions) to students who looked confused, who asked their neighboring student survey questions, and who raised their hand. They also spared no effort to remind participants not to ask their peers’ ratings to ensure confidential and independent responses. Consistency among the four was maintained through regular communication, coordination, and adherence to ethical guidelines, ensuring a cohesive and reliable approach to assisting the children and collecting data.

### 2.3. Measures and Variables

#### 2.3.1. Overall Leisure Enjoyment (Independent Variable)

Children’s overall leisure enjoyment was measured using the Children’s Assessment of Participation and Enjoyment (CAPE). The CAPE consists of 55 items capturing diversity and intensity of participation, contexts (where and with whom activities occurred), and perceived enjoyment across five leisure activity domains: recreational (12 items), physical (13 items), social (10 items), skill-based (10 items), and self-improvement (10 items) activities outside of school requirements. For the current study, only the enjoyment ratings were used. Children rated enjoyment on a 5-point Likert scale ranging from Not at all (1), Somewhat/Sort of (2), Pretty much (3), Very much (4), to Love it (5). For each domain, enjoyment scores were calculated by averaging across activities the child actually engaged in. An overall leisure enjoyment score was then derived by averaging the five domain scores.

#### 2.3.2. Emotional Self-Efficacy (ESE, Mediator)

Self-efficacy reflects an individual’s belief in their capability to organize and execute actions required to manage situations and achieve desired outcomes [26]. Emotional self-efficacy (ESE), a subdomain of self-efficacy, pertains to one’s perceived ability to regulate and effectively cope with negative emotions such as sadness, anger, or anxiety. In the present study, ESE was measured using the emotional subscale of the Self-Efficacy Questionnaire for Children (SEQ-C) [27]. This subscale comprises eight items rated on a 5-point Likert scale (1 = Not at all to 5 = Very well), with higher mean scores indicating greater emotional self-efficacy. The internal consistency of the emotional subscale in this sample was acceptable (α = 0.69).

#### 2.3.3. Life Satisfaction (Dependent Variable)

Subjective well-being (SWB) is defined as an individual’s comprehensive evaluation of life quality, encompassing both affective and cognitive dimensions [28]. Life satisfaction represents the cognitive dimension of SWB, reflecting a child’s appraisal of their overall life circumstances [29]. In this study, life satisfaction—measured using the Student Life Satisfaction Scale (SLSS)—served as a proxy indicator of SWB, consistent with prior research on child well-being [29]. The SLSS items are rated on a 6-point scale (Strongly Disagree = 1 to Strongly Agree = 6) reflecting well-being over the past several weeks [29]. Two negatively worded items were reverse-coded before computing the mean total score. The SLSS demonstrated good internal consistency in the current sample (α = 0.82).

Given the documented reliability and validity of the SEQ-C and the SLSS, as well as the study participants’ grade-level reading ability, the English version of both assessments was administered with language supports provided as needed. To strengthen temporal ordering for the mediation model, the CAPE was administered first. Two months later, the SEQ-C and SLSS were administered concurrently. This design supported the hypothesized pathway from overall leisure enjoyment to emotional self-efficacy to life satisfaction. Ten students were absent during the CAPE administration, and fifteen were absent during the SEQ-C and SLSS administration, all for excusable reasons (e.g., illness).

### 2.4. Data Preparation and Analysis

All data were inspected and processed prior to statistical analysis to ensure accuracy and integrity. First, the dataset was screened for missing values, implausible values, and data entry errors. Non-numeric entries (e.g., “.” or blanks) within numeric fields were recorded as missing (NA) to allow for proper handling in R. Descriptive checks (e.g., frequency tables and histograms) were conducted to identify potential outliers or extreme scores. Outliers were examined for data entry errors and retained if plausible, given the sample population.

Second, cases with missing or invalid data on key variables (IV, mediator, or DV) were excluded using listwise deletion as the mediation model requires complete observations. The final analytic sample included *n* = 77 valid, complete observations. This approach was selected to ensure consistency across models, though it inherently reduces sample size and statistical power. Because listwise deletion assumes data are missing completely at random, violation of this assumption may introduce bias if the excluded cases systematically differ from retained participants. To assess the robustness of the mediation results, a sensitivity analysis using multiple imputations (via the mice package in R) was conducted. The imputed dataset yielded similar patterns of direct and indirect effects, suggesting that the findings were generally stable despite the missing data.

Descriptive statistics were computed using all available data for each variable separately with psych package in R. In contrast, the mediation analysis applied listwise deletion, which required complete data across the independent, mediator, and dependent variables, and was conducted using the mediation package. The analysis estimated the Average Direct Effect (ADE) of overall leisure enjoyment on life satisfaction, the Average Causal Mediation Effect (ACME) through emotional self-efficacy, and the Total Effect combining both direct and indirect pathways. Nonparametric bootstrapping with 500 simulations was used to generate percentile-based 95% confidence intervals. A significance threshold of *p* < 0.05 was applied to all inferential analyses.

## 3. Results

### 3.1. Descriptive Statistics

Descriptive statistics and bivariate correlations are shown in Table 1. The dependent variable was positively correlated with both the mediator and the independent variable.

### 3.2. Mediation Analysis

**Figure 1:**
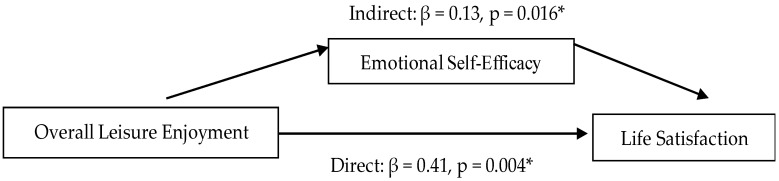
Final Mediation Analysis Model of Three Variables. * *p* < 0.05.

The mediation analysis results indicated that the total effect of overall leisure enjoyment on life satisfaction was statistically significant (β = 0.54, 95% CI [0.23, 0.90], *p* = 0.004). The direct effect remained significant after accounting for the mediator (β = 0.41, 95% CI [0.15, 0.73], *p* = 0.004). The indirect effect through emotional self-efficacy was also statistically significant (β = 0.13, 95% CI [0.03, 0.29], *p* = 0.016), indicating that approximately 25% of the total effect was mediated (95% CI [0.05, 0.52], *p* = 0.012). These findings support the hypothesized mediating role of emotional self-efficacy (as shown in Figure 1), suggesting that children who experience greater enjoyment in leisure activities are more likely to report higher emotional self-efficacy, which in turn contributes to greater life satisfaction.

## 4. Discussion

This study examined whether emotional self-efficacy mediates the relationship between children’s positive leisure experiences and their subjective well-being. The results suggest that children’s perceived enjoyment of activities outside of school has a direct influence on their well-being. Also, emotional self-efficacy significantly mediated the association between overall leisure enjoyment and life satisfaction, accounting for approximately one-quarter of the total effect. Taken together, these findings hold important implications for pediatric mental health, particularly within marginalized communities where children often encounter heightened stressors, limited access to recreational opportunities, and reduced support for developing emotional self-efficacy.

### 4.1. Interpretation of Findings

The significant indirect effect demonstrates that children who derive greater enjoyment from leisure activities tend to perceive themselves as more capable of managing emotions such as sadness, frustration, or worry, which in turn contributes to greater life satisfaction. This finding aligns with prior work emphasizing the immediate affective benefits of enjoyable physical activity participation, such as fostering confidence [19] and intrinsic motivation [7,13,14]. In addition, this pattern is consistent with Fredrickson’s broaden-and-build theory of positive emotions [24], which posits that positive experiences expand individuals’ cognitive and behavioral repertoires and, over time, cultivate enduring personal resources such as emotional self-efficacy. Enjoyment may therefore operate as a proximal positive emotion that enables children to internalize adaptive coping beliefs, reinforcing their perceived ability to regulate emotions and handle everyday challenges.

Notably, the direct path from leisure enjoyment to life satisfaction remained significant, suggesting that the affective and social benefits of engaging in enjoyable leisure activities extend beyond self-efficacy beliefs. The immediate pleasure, social connectedness, and sense of autonomy that accompany leisure participation may independently elevate children’s subjective well-being. Thus, both mechanisms—direct experiential gratification and indirect resource building through emotional self-efficacy—jointly explain how leisure enjoyment contributes to overall life satisfaction.

Beyond confirming this theoretical linkage, the current findings extend the broaden-and-build theory in several meaningful ways. First, they demonstrate that the theory applies not only to adults’ discrete positive emotions but also to children’s naturally occurring experiences of leisure enjoyment—micro-moments of joy and engagement that collectively shape emotional self-beliefs. This suggests that the process of building psychological resources can emerge through the ordinary rhythms of play and leisure, not solely through structured achievement or mastery experiences. Second, the persistence of a significant direct effect from leisure enjoyment to life satisfaction underscores that positive emotions can enhance well-being through both immediate experiential pathways (e.g., pleasure, social connectedness, autonomy) and developmental resource-building pathways (e.g., emotional self-efficacy). Together, these dual mechanisms together reinforce the theoretical view that positive emotions serve both transient and cumulative functions in human development.

### 4.2. Contextual Significance

The strength of both direct and indirect effects may reflect the sociocultural context of this sample—elementary school children residing in a predominantly Hispanic, economically marginalized U.S.–Mexico border community. Within such environments, children often face constrained access to structured extracurricular programs, limited neighborhood safety, and restricted availability of recreational spaces [2,5,6]. These contextual barriers make opportunities for enjoyable leisure experiences (through informal play, creative expression, or social interaction) especially meaningful, as they may represent one of the few consistent sources of positive emotion, intrinsic reward, and self-determined activity in children’s daily lives.

Viewed through the lens of the broaden-and-build theory of positive emotions [24], this interpretation highlights an equity-relevant dimension of the framework. Enjoyable leisure activities may operate as a natural context in which children accumulate positive affective experiences that, over time, build coping confidence and emotional resilience despite limited structural support. In marginalized settings, these micro-moments of joy, autonomy, and social connection can act as psychological buffers against contextual stressors, helping children sustain well-being under adversity. Moreover, Hispanic cultural values such as familismo—the prioritization of family cohesion, loyalty, and mutual support—can further amplify the emotional salience of shared enjoyment and reinforce children’s sense of connectedness and belonging [3,4].

### 4.3. Developmental Considerations

From a developmental perspective, the present findings indicate that even late-childhood participants (approximately 10–12 years) are capable of linking affective experiences to emerging self-beliefs about emotional competence. This extends earlier evidence that younger children may experience enjoyment as an immediate reward without consciously connecting it to self-efficacious beliefs [30]. The significant mediation observed here may therefore reflect the transition toward more reflective self-understanding in preadolescence.

Taken together, these findings underscore the “so what” of the study: cultivating accessible, enjoyable leisure opportunities is not merely a lifestyle matter but a theoretically grounded, equity-promoting mechanism for building children’s emotional resources and advancing mental well-being.

### 4.4. Study Limitations and Directions for Future Research

Several methodological and interpretive limitations should be acknowledged.

#### 4.4.1. Sample Size and Statistical Power

The study sample size (*n* = 77) remains modest for mediation modeling. Small-to-moderate indirect effects generally require larger samples for stable estimation, even with bootstrapped confidence intervals. Therefore, the observed significant mediation should be interpreted as preliminary yet promising evidence rather than definitive confirmation. Future research employing larger, adequately powered samples will be critical to validate these findings and to enable the examination of potential moderators such as gender, grade level, or cultural values (e.g., familismo).

#### 4.4.2. Measurement Reliability

The internal consistency of the emotional self-efficacy subscale of the Self-Efficacy Questionnaire for Children (Cronbach’s α = 0.69) was acceptable but slightly below the optimal range (0.70–0.80). Limited reliability can attenuate observed correlations and underestimate mediation strength. The detection of a significant indirect effect despite this modest reliability suggests a robust underlying relationship. Nonetheless, future studies should consider refining or culturally adapting the SEQ-C emotional subscale to improve psychometric precision, especially for bilingual or bicultural child populations.

#### 4.4.3. Cross-Sectional Research Design

The cross-sectional nature of this study restricts causal inference. Although the order of data collection (CAPE administered before SEQ-C and SLSS) was designed to strengthen temporal plausibility, true temporal mediation cannot be established without longitudinal data. Future longitudinal or experimental designs could clarify whether leisure enjoyment predicts later increases in emotional self-efficacy, which subsequently enhance life satisfaction over time.

#### 4.4.4. Self-Report Bias and Contextual Constraints

All data was collected via self-report measures, which may be influenced by social desirability or comprehension challenges, especially among younger participants. While extensive support (e.g., verbal explanations and supervision) was provided to reduce misunderstanding, self-report data still rely on children’s introspective ability to evaluate internal states. Additionally, the study’s single-site sampling within a U.S.–Mexico border community limits generalizability to children in other cultural or socioeconomic contexts. Multi-site studies that capture greater environmental and cultural diversity would allow for stronger external validity.

#### 4.4.5. Scope of the Model

The statistical model focused on emotional self-efficacy as the sole mediator. Children’s well-being, however, is multifaceted and may also be shaped by other contributing factors such as perceived independence in self-care, academic achievement, autonomy, social support, and family connectedness. Incorporating these constructs in future models could provide a more comprehensive understanding of how positive leisure experiences influence well-being across multiple developmental constructs.

#### 4.4.6. Directions for Future Research

Taken together, future research should aim to replicate this study with larger and more diverse samples of children and youth (e.g., including both urban and rural Hispanic communities and diverse socioeconomic backgrounds). Moreover, future studies should also examine how structural inequities—such as poverty, neighborhood safety, and access to community programs—shape both children’s opportunities for enjoyable leisure engagement and their subsequent mental health outcomes. It would also be valuable to test whether structured leisure programs that intentionally integrate emotion-regulation skill building can enhance both enjoyment and self-efficacy outcomes. Lastly, future studies can integrate qualitative interviews to capture children’s lived experiences of leisure participation and emotional growth in context.

### 4.5. Implications for Pediatric Practice

These findings underscore the importance of prioritizing children’s access to enjoyable leisure activities as a direct means of supporting mental health and subjective well-being. For children living in marginalized communities, where structural barriers such as poverty, limited recreational resources, and reduced recreational program availability are present, intentionally promoting accessible and enjoyable activity engagement becomes especially critical.

Pediatric practitioners, including occupational therapists, physical therapists, and speech-language pathologists, along with educators and social workers, can play a pivotal role in facilitating enjoyable activity participation. They can advocate for inclusive community programs, integrate various activity opportunities into school and after-school settings, and collaborate with community organizations to design programs that are culturally responsive and sensitive to the realities of underserved populations. Incorporating enjoyable activities with social–emotional learning opportunities that strengthen children’s self-regulation skills offers a more holistic, equity-oriented approach to pediatric health promotion [31].

## 5. Conclusions

This study demonstrated that children’s enjoyment of leisure activities was strongly and positively associated with life satisfaction, and that this relationship was partially mediated by emotional self-efficacy. These findings align with Fredrickson’s broaden-and-build theory of positive emotions, illustrating how positive activity experiences can expand children’s emotional resources and subjective well-being. Supporting children’s participation in enjoyable leisure activities, alongside opportunities to build social–emotional skills, provides a balanced and integrative approach to fostering health and well-being. Within marginalized communities, such efforts may serve as a vital strategy for addressing mental health disparities and advancing equity in childhood well-being.

This research contributes to scientific literature in several keyways. It advances the understanding of how positive leisure experiences influence children’s subjective well-being by clarifying the relative influence of direct and indirect pathways. Additionally, it is one of the first studies to explore positive leisure experiences in a sample of elementary school children from a predominantly Hispanic background as well as a marginalized community. Unlike much of the previous research that focuses on physical activities, this study uniquely examined a broader range of leisure activities, including recreational, social, skill-based, and self-improvement pursuits.

## Figures and Tables

**Table 1:** Descriptive Statistics and Correlations.

Variable	N	M	SD	1	2	3
1. Overall Leisure Enjoyment	90	3.89	0.81	-		
2. Emotional Self-Efficacy	85	3.06	0.76	0.22	-	
3. Life Satisfaction	85	4.38	1.21	0.35 *	0.45 *	-

Note. Descriptive statistics in Table 1 are based on all available non-missing observations for each variable individually. The mediation analysis used listwise deletion across these variables, resulting in a final analytic sample of 77 complete cases. * *p* < 0.05.

## Data Availability

The data presented in this study are available on request from the corresponding author due to ethical reasons for the restriction.
